# A Radiomics Nomogram Integrated With Clinic-Radiological Features for Preoperative Prediction of DNA Mismatch Repair Deficiency in Gastric Adenocarcinoma

**DOI:** 10.3389/fonc.2022.865548

**Published:** 2022-07-01

**Authors:** Yahan Tong, Jiaying Li, Jieyu Chen, Can Hu, Zhiyuan Xu, Shaofeng Duan, Xiaojie Wang, Risheng Yu, Xiangdong Cheng

**Affiliations:** ^1^ Department of Radiology, The Cancer Hospital of the University of Chinese Academy of Sciences (Zhejiang Cancer Hospital), Hangzhou, China; ^2^ Institute of Basic Medicine and Cancer (IBMC), Chinese Academy of Sciences, Hangzhou, China; ^3^ Key Laboratory of Prevention, Diagnosis and Therapy of Upper Gastrointestinal Cancer of Zhejiang Province, Hangzhou, China; ^4^ Department of Radiology, The First Clinical Medical College of Zhejiang Chinese Medical University, Hangzhou, China; ^5^ Department of Radiology, The First Affiliated Hospital of Zhejiang Chinese Medical University, Hangzhou, China; ^6^ Department of Gastric Surgery, The Cancer Hospital of the University of Chinese Academy of Sciences (Zhejiang Cancer Hospital), Hangzhou, China; ^7^ Precision Health Institution, GE Healthcare, Shanghai, China; ^8^ Department of Radiology, Second Affiliated Hospital, Zhejiang University School of Medicine, Hangzhou, China

**Keywords:** gastric cancer/adenocarcinoma, radiomics, tomography, X-ray computed, nomogram, DNA mismatch repair deficiency

## Abstract

**Purpose:**

To develop and validate a radiomics nomogram integrated with clinic-radiological features for preoperative prediction of DNA mismatch repair deficiency (dMMR) in gastric adenocarcinoma.

**Materials and Methods:**

From March 2014 to August 2020, 161 patients with pathologically confirmed gastric adenocarcinoma were included from two centers (center 1 as the training and internal testing sets, n = 101; center 2 as the external testing sets, n = 60). All patients underwent preoperative contrast-enhanced computerized tomography (CT) examination. Radiomics features were extracted from portal-venous phase CT images. Max-relevance and min-redundancy (mRMR) and least absolute shrinkage and selection operator (LASSO) methods were used to select features, and then radiomics signature was constructed using logistic regression analysis. A radiomics nomogram was built incorporating the radiomics signature and independent clinical predictors. The model performance was assessed using receiver operating characteristic (ROC) curve analysis, calibration curve, and decision curve analysis (DCA).

**Results:**

The radiomics signature, which was constructed using two selected features, was significantly associated with dMMR gastric adenocarcinoma in the training and internal testing sets (*P* < 0.05). The radiomics signature model showed a moderate discrimination ability with an area under the ROC curve (AUC) of 0.81 in the training set, which was confirmed with an AUC of 0.78 in the internal testing set. The radiomics nomogram consisting of the radiomics signature and clinical factors (age, sex, and location) showed excellent discrimination in the training, internal testing, and external testing sets with AUCs of 0.93, 0.82, and 0.83, respectively. Further, calibration curves and DCA analysis demonstrated good fit and clinical utility of the radiomics nomogram.

**Conclusions:**

The radiomics nomogram combining radiomics signature and clinical characteristics (age, sex, and location) may be used to individually predict dMMR of gastric adenocarcinoma.

## Introduction

Globally, gastric cancer (GC) is one of the most common malignant tumors and is a common cause of cancer-related death (1, 2). The symptoms of early GC are occult and often neglected, so many patients in China have locally advanced disease at the time of diagnosis (3). Since microsatellite instability (MSI) was found in hereditary non-polyposis colorectal cancer in 1993, it has been detected in many forms of malignant tumor, such as lung and bladder cancers (4–6). Increasingly, clinical trials have confirmed that MSI/DNA mismatch repair deficiency (dMMR) plays an important role in the occurrence and prognosis of GC (7–9). The Cancer Genome Atlas has identified MSI or dMMR as a hallmark of the second molecular subtype of GC (10, 11). MSI or dMMR status in GC is crucial for clinical decision making, as it identifies patients with different treatment responses and prognoses of GC (12–14). According to the 2021 guidelines of the National Comprehensive Cancer Network (NCCN) for GC (15), all newly diagnosed GC patients should be tested for MSI by polymerase chain reaction (PCR)-based molecular testing or DNA mismatch repair (MMR) protein using immunohistochemistry (IHC). Conventional MSI/MMR testing is recommended, but many patients remain untested. Testing for MSI/MMR is expensive, and interobserver variability in interpretation has been found among the different primary modalities (16, 17). Presurgery prediction of mismatch repair gene expression in GC would be of great significance for the selection of the treatment plan and treatment method and the evaluation of prognosis. There is a critical need for development of an objective, broadly accessible, and cost-efficient testing method for patients with GC.

Radiomics can provide more information than conventional CT images. The rise of radiomics makes it possible to convert imaging data into high-dimensional feature data, and the multiple quantitative features extracted from original images by bioinformatics can predict the underlying biological behavior of tumors (18–20). In recent years, many studies have found that certain radiomics features have diagnostic and prognostic value (21–23). Zhang et al. reported that the magnetic resonance imaging (MRI) texture signature may serve as a potential predictive biomarker for immunophenotyping and overall survival of intrahepatic cholangiocarcinoma patients (23). In the field of radio-genomics, imaging features are allied to genotype. Tumors with poor prognosis also tend to have greater genomic heterogeneity of tumor tissues (24). Radio-genomics is an evolution on the foundation of radiomics, which assumes that genomic heterogeneity at the microscopic level can present as tumor heterogeneity, and variation in the microenvironment of the lesion may be manifested as morphological characteristics and macroscopic images (25). Hence, the application of radiomics offers a new path to remove the limitations of traditional biopsy methods. Kim et al. found that the texture features based on multiparametric MRI were particularly connected with the isocitrate dehydrogenase mutation and tumor aggressiveness in diffuse lower-grade glioma (26). In recent years, radiomics nomograms, which are based on multiple variables, have been widely accepted as a user-friendly tool for predicting prognosis and have been used successfully to forecast the genotype of malignant tumors preoperatively (27–29). Wang et al. reported that the radiomics nomogram integrated with clinic-radiological features holds promise for clinical use as a non-invasive tool in the individual prediction of lymph node metastasis in GC (30). Wang et al. found that the nomogram-integrated CT-radiomics signature and CT-reported T stage can enhance prediction of the human epidermal growth factor receptor 2 status of esophagogastric junction adenocarcinoma before surgery (31).

Therefore, in this research, we aimed to develop and validate a radio-clinical nomogram based on a combination of radiomics signature and clinical risk factors for the preoperative prediction of DNA mismatch repair deficiency in patients with gastric adenocarcinoma.

## Materials and Methods

### Patients

**Figure 1:**
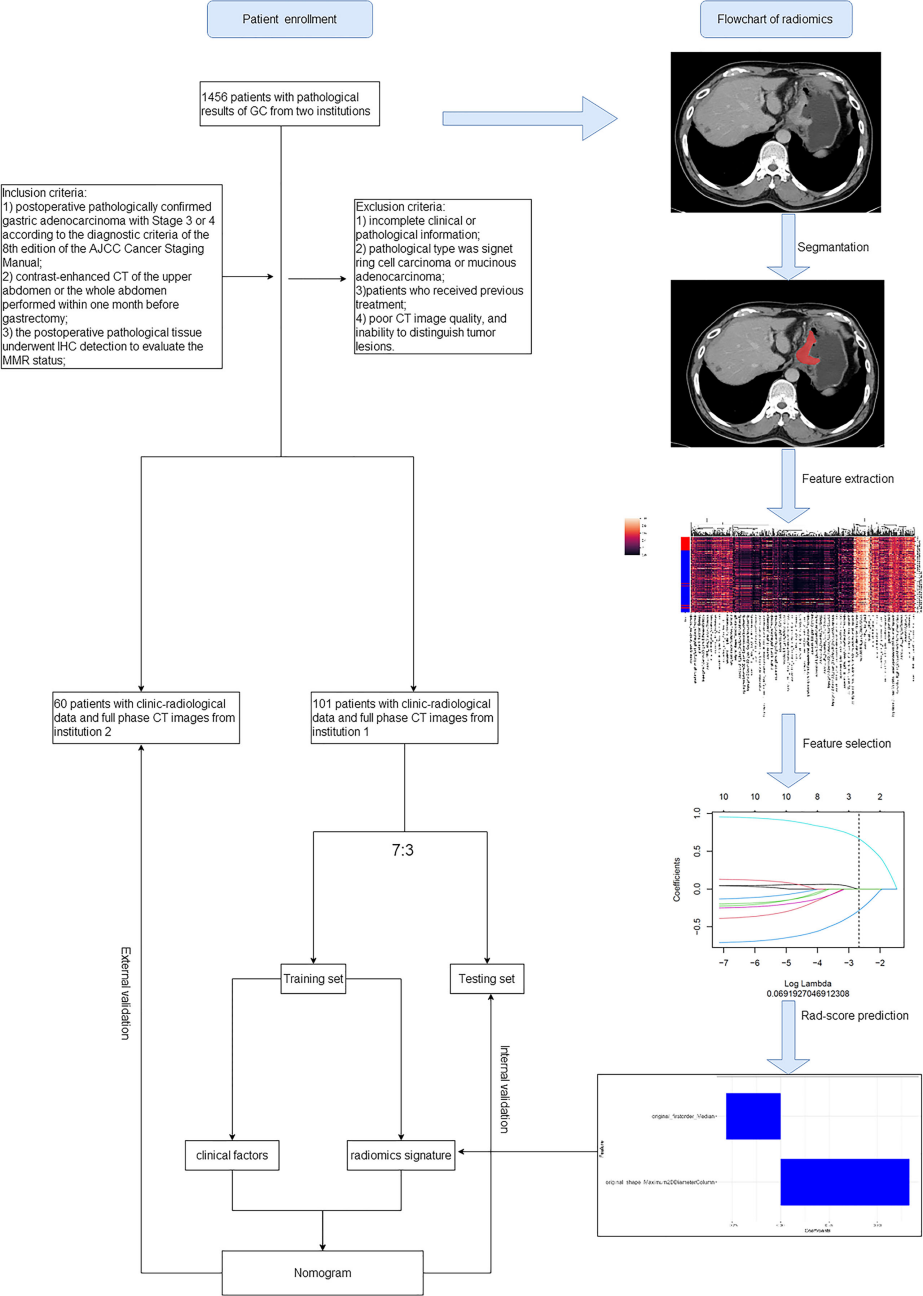
Flowchart of the recruiting study population and model construction.

This retrospective study was approved by the review board of our institution (The Cancer Hospital of the University of Chinese Academy of Science). The requirement for informed consent was waived. This study retrospectively collected data from 1,456 patients with pathologically confirmed GC who underwent radical gastrectomy between March 2014 and August 2020 at two centers. In total, 161 patients were enrolled according to the inclusion and exclusion criteria (detailed below). Among these, 101 cases from center 1 (The Cancer Hospital of the University of Chinese Academy of Science) were used as the training and internal testing sets, and 60 cases from center 2 (The Second Affiliated Hospital Zhejiang University School of Medicine) were used as the external testing set. A flowchart of the patient record selection process is shown in **Figure 1**. All patients underwent preoperative contrast-enhanced CT examination of the abdomen.

The inclusion criteria were as follows: (1) postoperative pathologically confirmed gastric adenocarcinoma at stage 3 or 4 according to the diagnostic criteria of the 8th edition of the American Joint Committee on Cancer Staging Manual; (2) patients who underwent contrast-enhanced CT of the upper abdomen or the whole abdomen within 1 month before surgery; (3) IHC detection was performed on pathological tissue to evaluate the MMR status postoperatively. The exclusion criteria were the following: (1) incomplete clinical or pathological information (2); pathological type signet ring cell carcinoma or mucinous adenocarcinoma; (3) treatment was given before surgery; (4) poor CT image quality with longest diameter of less than 5mm. Patients’ clinical and imaging data including sex, age, tumor location, and MMR status were recorded. The location of GC was based on pathology, including cardia, gastric body, and gastric antrum.

### CT Image Acquisition

All patients underwent contrast-enhanced abdominal CT using the following multidetector row CT systems: BrightSpeed, Optima CT680 Series (GE Medical Systems), and Siemens Somatom Definition AS 64, Perspective (Siemens Medical Systems). The acquisition parameters were as follows: tube voltage, 120–130 kV; tube current, 150–300 mAs; reconstructed axial-section thickness 5 mm, slice interval 5 mm, pitch 0.6. The contrast agents were Ultravist (Bayer Schering Pharma, Berlin, Germany), Optiray (Liebel-Flarsheim Canada Inc., Kirkland, Quebec, Canada), and Iohexol (Beijing North Road Pharmaceutical Co. Ltd., Beijing, China). A total of 70–100 ml of contrast agent was administered using a pump injector into an antecubital vein. Arterial phase and portal venous phase contrast-enhanced CT scans were performed after delays of 30–35 s and 50–60 s after injection of the contrast medium, respectively.

### Mismatch Repair Protein Status

IHC was used to evaluate the results of MMR protein status according to the 2021 Gastric Cancer NCCN guidelines as follows: FmutL homologue 1 (MLH1), mutS homologue 2 (MSH2), mutS homologue 6 (MSH6), and PMS1 homologue 2 (PMS2) proteins were detected, which were positively located in the nucleus. Any protein expression loss was evaluated as dMMR (mismatch repair function defect), and all four protein expressions were positive as pMMR (mismatch repair function complete).

### Tumor Segmentation

**Figure 2:**
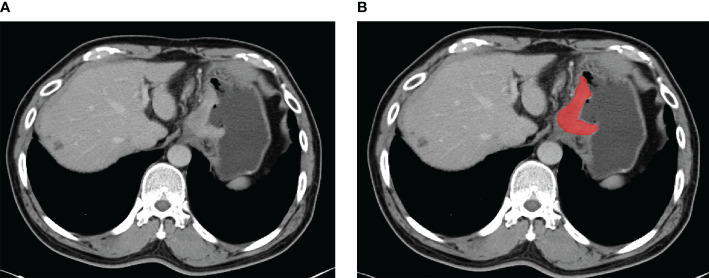
An example of manual segmentation in gastric cancer. **(A)** Localized thick wall of gastric cancer with enhancement is observed on the portal venous phase computed tomography (CT) image. **(B)** Manual segmentation on the same axial slice is depicted with red label.

The portal-venous phase CT images of GC patients were acquired from the picture archiving and communication systems. The patient’s abdominal portal venous phase CT digital image was exported in digital imaging and communications in medicine (DICOM) format. Radiologists with over 5 years of experience in interpreting abdominal diseases examined each layer of the patients’ CT images. Two radiologists outlined the regions in each patient’s CT images. Lesions were delineated using ITK-SNAP (version 3.8.0, http://www.itksnap.org) as shown in **Figure 2**.

For the tumor regions of interest (ROIs), radiologists reviewed all of each patient’s CT image slices and selected the largest tumor area slice to segment. The ROI was selected to cover the whole area of the tumor. Observer 1 delineated the lesions of all patients with GC. Observer 2 confirmed the tumor segmentation (32). If the segmented lesions were inconsistent between the two observers, consensus was reached by discussion. During the delineation process, ROI selection avoided the areas of gastric air, necrosis, and adipose tissue.

### Radiomics Feature Extraction and Selection

The radiomics feature extraction process for this study was performed using YITU AI Enabler, which is an integrated machine learning platform for medical data analysis using well-established python pyradiomics (version 3.0.1) and the scikit-learn (version 0.22) package. Resampling through the radiomics features was first extracted based on the original image data set. Then a feature stability check was performed on the features extracted within the lesion ROI and the extended lesion ROI to filter out unstable features with minor change of ROI using an intra-class correlation algorithm. The extended lesion ROI was made by extending the boundary of lesion ROI by one image pixel. We used max-relevance and min-redundancy (mRMR) and least absolute shrinkage and selection operator (LASSO) methods to select features, and then the rad-score of each GC patient was calculated by their coefficients.

### Construction of a Predictive Model

Multivariable logistic regression analysis was used to develop a prediction model by combining significant rad-score, sex, age, and tumor location (with *P* values less than 0.05 in the univariable analysis). In the training set, for clinicians’ convenience we constituted the model as a radio-clinical nomogram based on multivariable logistic regression analysis. Finally, the generalization ability of the nomogram was evaluated in the internal and external testing sets.

### Performance of the Radiomics Nomogram

The predictive performance of the radiomics nomogram was evaluated using the receiver operator characteristic (ROC) curve, calibration curve, and decision curve analysis (DCA). Model evaluation 10-fold cross-validation was used in model training, and the diagnostic performance of radiomics, clinical, and radio-clinical models were validated in the internal testing set. The area under the ROC curve (AUC), sensitivity, specificity, accuracy, positive predictive value (PPV), and negative predictive value (NPV) of the nomogram were calculated. DCA analysis was performed to assess the model’s clinical utility by calculating the net benefits at different threshold probabilities. Finally, generalization of the radiomics nomogram was evaluated in the independent external testing set.

### Statistical Analysis

All statistical analyses were performed using R software (version 3.4.1; http://www.Rproject.org) and IBM SPSS Statistics (Version 26.0; IBM Corp., New York, USA). Quantitative data were described by mean ± standard deviation, and qualitative data by frequency (percent). Normally distributed continuous data were compared using the Student’s t-test. The chi-square test was used to compare the distribution of categorical data between groups. A multivariate logistic regression analysis was applied to determine the independent predictors among all the clinical variables. P < 0.05 was considered statistically significant. The “glmnet” package was used for LASSO logistic regression analysis. The multivariable logistic regression analysis and calibration plots were conducted using the “rms” package. The ROC plots of radiomics signature were performed with the “pROC” package. The “rmda” package was applied for decision curve analysis (DCA).

## Results

### Clinical Characteristics

Among 101 patients with GC from center 1, there were dMMR (n = 35) and pMMR (n = 66) cases. The patients were randomly divided into a training set of 71 cases and an internal testing set of 30 cases. In the training set, statistically significant differences in sex, age, and tumor location were found between dMMR and pMMR GC patients (*P* < 0.05). In the training and internal testing sets, a significantly higher rad-score was found in dMMR than in pMMR in both cohorts (*P* < 0.05). Among 60 patients with GC from center 2 as an external testing set, there were dMMR (n = 21) and pMMR (n = 39) cases. Additional details are provided in **Table 1**.

**Table 1:** Clinic-radiological characteristics of patients in the training and testing sets.

Characteristic	Training set		Internal testing set		External testing set	
	dMMR	pMMR	dMMR	pMMR	dMMR	pMMR
**Age (Y)**						
mean (sd)	72.8 (9.1)	65.6 (10.4)	69.3 (7)	68.1 (8.1)	70.0 (8.5)	64.8 (9.3)
**Sex**						
Male	13 (54.2)	38 (80.9)	6 (54.5)	15 (78.9)	9 (42.9)	33 (84.6)
Female	11 (45.8)	9 (19.1)	5 (45.5)	4 (21.1)	12 (57.1)	6 (15.4)
**Location**						
Cardia	1 (4.2)	14 (29.8)	1 (9.1)	7 (36.8)	17 (81.0)	12 (30.8)
Gastric body	10 (41.7)	19 (40.4)	5 (45.5)	7 (36.8)	3 (14.3)	21 (53.8)
Antrum	13 (54.2)	14 (29.8)	5 (45.5)	5 (26.3)	1 (4.7)	6 (15.4)
**Rad-sore**						
Median [iqr]	-0.2 [-0.7, 0.6]	-1.1 [-1.5,-0.8]	-0.6 [-0.8, 0.0]	-1.1 [-1.3,-0.7]	-1.1 [-1.4,-0.8]	-1.5 [-1.9,-1.0]

### Radiomics Feature Selection and Radiomics Signature Construction

**Figure 3:**
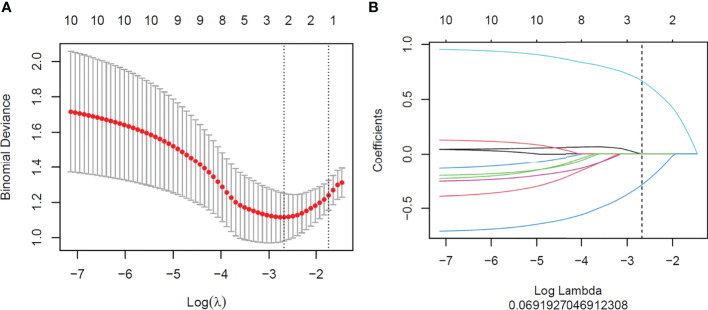
Feature selection with the least absolute shrinkage and selection operator (LASSO) binary logistic regression model. **(A)** Tuning parameter (λ) selection of the LASSO model. Binomial deviance was drawn versus log(λ). Vertical dotted lines were plotted at the best value using 10-fold cross-validation to tune parameter (λ) selection in the LASSO model. **(B)** LASSO coefficient profiles of the features. Each colored line represents the corresponding coefficient of each feature. A vertical dotted line was drawn at the selected λ, where non-zero coefficients were obtained with two features.

A total of 1,648 radiomics features were extracted from CT images of each GC patient, among which 989 features with good stability were selected for radiomics model establishment. Initially, mRMR was performed to eliminate the redundant and irrelevant features, and 30 features were retained. Then, LASSO was conducted to choose the optimized subset of features to construct the final model. The optimal λ in the LASSO logistic regression analysis with 10-fold cross-validation was used to select the best radiomics feature with a non-zero coefficient, as shown in **Figure 3**. Finally, two radiomics features were selected to construct the radiomics signature, and the rad-score was calculated by summing the selected features weighted by their coefficients. The final formula for rad-score is as follows:


Radscore=0.666∗original_shape_Maximum2DDiameterColumn+−0.283∗original_firstorder_Median + −0.747

### Development of an Individualized Radiomics Nomogram

**Figure 4:**
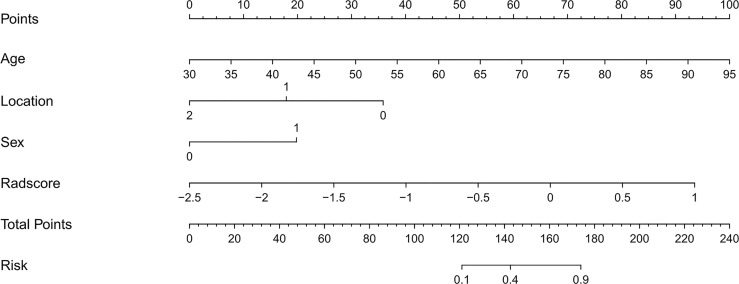
The CT-based radiomics nomogram. The radiomics nomogram was built in the training cohort, with the radiomics signature, sex (0 is male, 1 is female), age, and tumor location (0 is antrum, 1 is gastric body, 2 is cardia).

Univariate analysis showed that sex, age and tumor location with P values less than 0.05 were independent clinical risk factors for MMR status in GC patients. Multivariable analysis was performed to develop a prediction model by combining the rad-score, sex, age, and tumor location **(** **Table 2** **)**. Further, the radiomics nomogram is visualized in **Figure 4**. The formula for the nomoscore is as follows:


Nomoscore = (Intercept)∗−7.56566042486333+Age∗0.127948643930096+Location∗−1.49110528477808+Sex*1.64766133092359+Radscore∗2.22277808425775

**Table 2:** Univariate and multivariate logistic regression analysis of the clinic-radiological features.

Characteristics	Univariate analysis			Multivariate analysis		
	OR	95% CI	P value	OR	95% CI	P value
Age	1.08	[1.02;1.14]	<0.01	1.14	[1.03;1.25]	<0.01
Sex	3.57	[1.21;10.55]	0.021	5.19	[0.88;30.54]	0.068
Location	0.37	[0.17;0.79]	0.012	0.23	[0.07;0.73]	0.013
Rad score	5.51	[2.30;13.18]	<0.01	9.23	[2.95;28.92]	<0.01

### Performance of the Radiomics Nomogram

**Figure 5:**
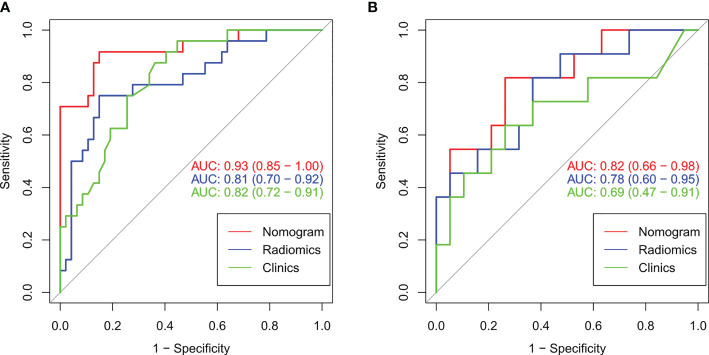
The ROC curves (AUC) of the three models in the training set **(A)** and internal testing set **(B)**.

**Figure 6:**
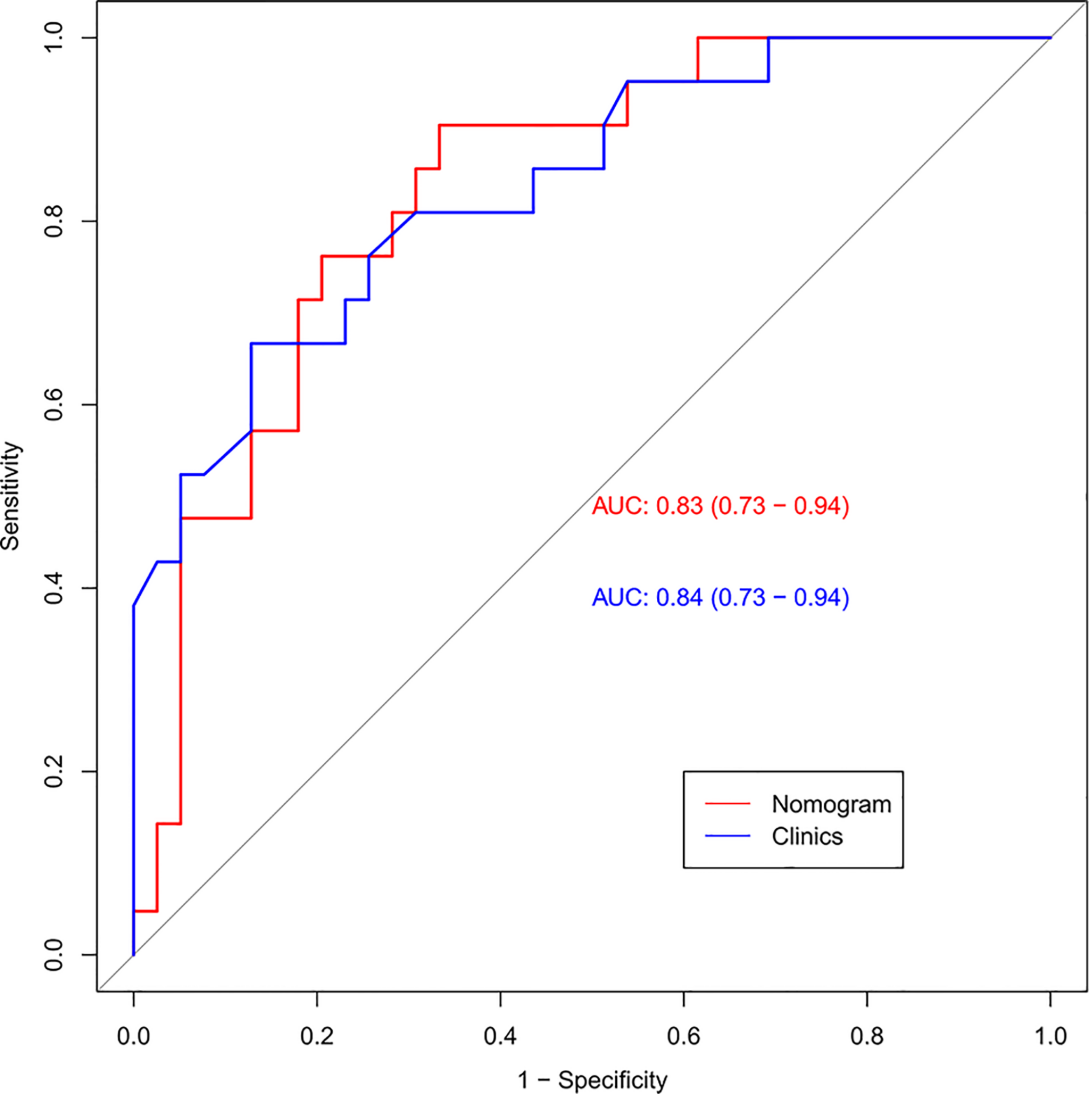
The ROC curves (AUC) of the external testing set.

**Figure 7:**
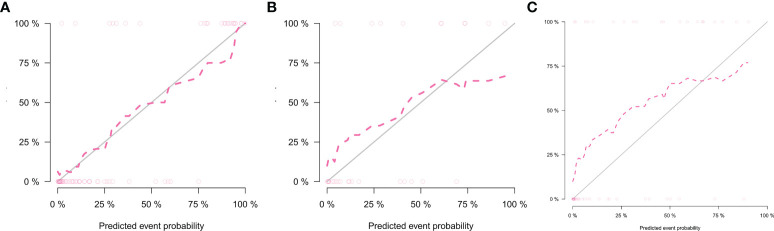
Calibration curves of the nomogram in the training set **(A)**, internal testing set **(B)**, and external testing set **(C)**.

**Figure 8:**
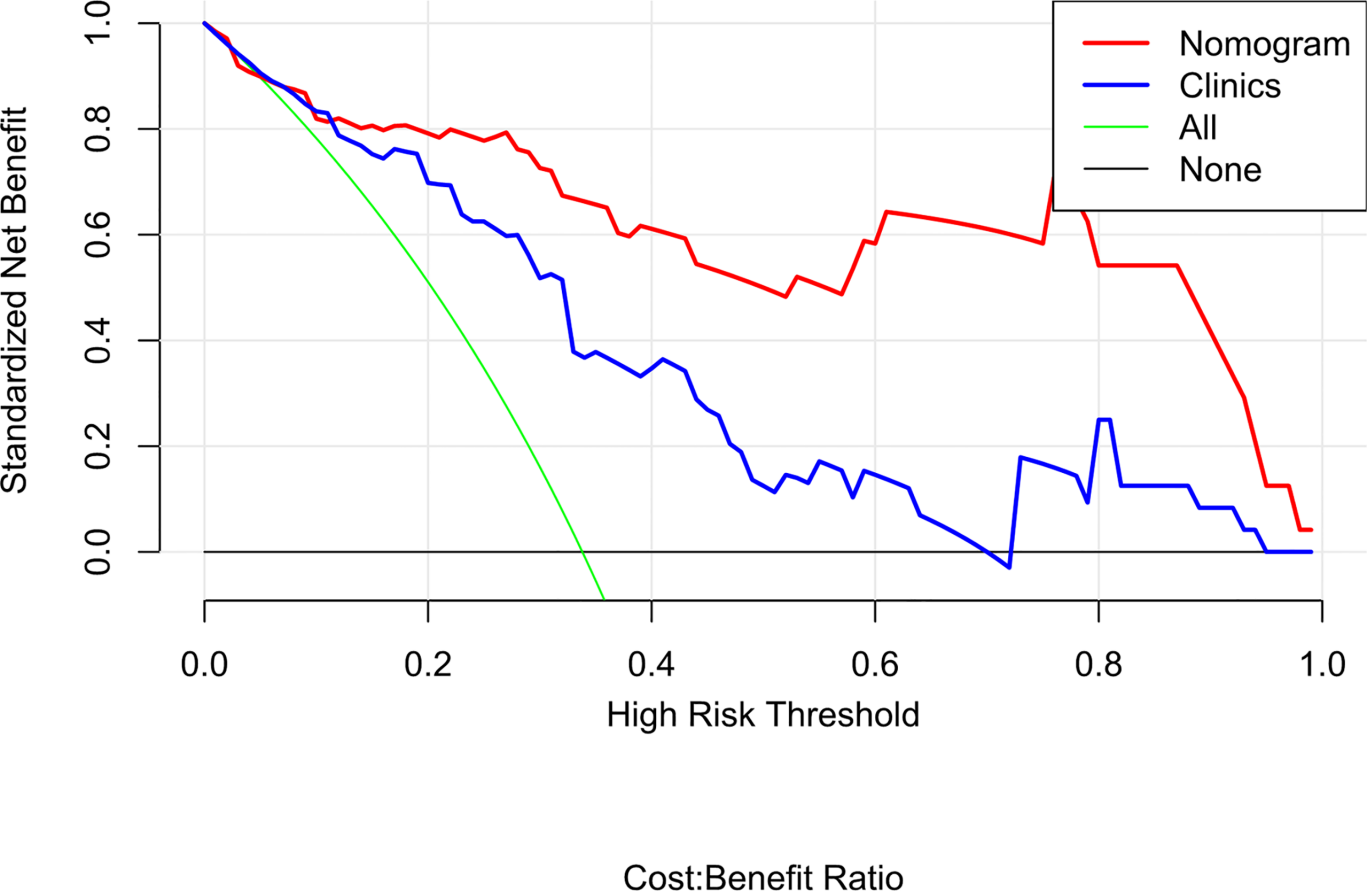
Decision curve analysis (DCA) for the radiomics nomogram and clinics model. The DCA indicated that more net benefits within the most of thresholds probabilities were achieved using the radiomics nomogram.

**Table 3** lists the performance of the radiomics nomogram in the training, internal, and external testing sets. The prediction model based on the radiomics features provided only moderate predictive power, as shown in **Figure 5**. The AUC value of radiomics signature in the training set and internal testing sets was 0.81 and 0.78, respectively. The predictive model based on clinical features alone showed that the AUC values in the training and internal testing sets were 0.82 and 0.69, respectively. The radiomics nomogram model combining clinical factors and radiomics features shows superior ability to differentiate MMR status compared with the other two models generated with clinical features and radiomics features alone. The AUC values of the radiomics nomogram in the training set and internal testing set were 0.93 and 0.82 **(** **Figure 5** **).** The external testing set radiomics nomogram showed an AUC value of 0.83 **(** **Figure 6** **)**. The calibration curve of the radiomics nomogram showed good predictions in both the training and validation cohorts **(** **Figure 7** **)**. The DCA of the radiomics nomogram demonstrated the higher overall net benefit compared to the clinics model, showing an excellent clinical utility in distinguishing MMR status (**Figure 8** **)**.

**Table 3:** Predictive performance of the radiomics nomogram.

Radiomics nomogram	AUC (95%CI)	Accuracy	Sensitivity	Specificity	PPV	NPV
**Training set**	0.93 (0.85–1.00)	0.873	0.917	0.851	0.759	0.952
**Internal testing set**	0.82 (0.66–0.98)	0.733	0.616	0.824	0.727	0.737
**External testing set**	0.83 (0.73–0.94)	0.767	0.821	0.667	0.821	0.667

## Discussion

In the present study, we developed and validated a radio-clinical nomogram for the prediction of the MMR status of GC perioperatively. The user-friendly nomogram, which consisted of the radiomics signature, sex, age, and tumor location, showed good performance in both cohorts and may effectively stratify patients according to MMR status. The combined analysis of multiple radiomics and clinical markers as a signature is the approach that demonstrates the most promise to change clinical practice (21, 33).

Since MSI was detected in many different types of tumors, the MMR status of tumors has become an important determinant in the choice of therapeutic method. In recent years, immunotherapy has gradually attracted attention and has developed rapidly. Immune checkpoint inhibitors, including anti-programmed death-1 and anti-cytotoxic T-lymphocyte-associated protein-4 antibodies, were effective for MSI-high or dMMR solid tumors in many trials (34). In 2017, the Food and Drug Administration of the United States approved pembrolizumab to treat patients with dMMR/MSI-H non-resectable or solid metastatic tumors. The MSI status is currently used as a biomarker for cancer immunotherapy (35). In addition, MMR status plays an important role in predicting the efficacy of neoadjuvant chemotherapy (13, 14). Accurate prediction of the DNA mismatch repair deficiency status is consequential for the selection of individualized treatment plans in patients with GC. A recent study found that deep learning can differentiate routine hematoxylin and eosin (H&E)-stained, formalin-fixed, paraffin-embedded digital whole-slide images (WSIs) of colorectal cancer into those with microsatellite stability and microsatellite instability, with an AUC of up to 0.84 (36). Rikiya et al. developed a deep learning model using 100 H&E-stained WSIs and found that they performed better than human experts (gastrointestinal pathologists) at detecting MSI in routine H&E-stained WSI (37). Some researchers have begun to use artificial intelligence to predict gene expression status non-invasively. The present study used more easily available imaging data and achieved good predictive performance. Radiomics enables non-invasive detection of the revealing relationship between invisible high-dimensional image features and pathophysiological characteristics. Radiomics has developed rapidly in recent years, and now more than 1,000 radiomics features are available for various aspects of tumor heterogeneity (38). The advantage of this study was presumably that it took radiomics scores, incorporating numerous quantitative features, into consideration. Radio-genomics builds on radiomics, which hypothesizes that genomic heterogeneity at the microscopic level may manifest in the tumor, and changes in the microenvironment within the tumor can be expressed on macroscopic images (18). Yang et al. reported that the proposed CT-based radiomics signature is associated with KRAS/NRAS/BRAF mutations; their study indicated that CT may be useful for the analysis of tumor genotype in colorectal cancer and thus helpful to determine therapeutic strategies (39). Combining analysis of clinical features and CT-based radiomics signature may improve predictive efficacy and allow patients to non-invasively choose individualized treatment plans (40).

In this study, dMMR accounted for only 8 percent of GC, a cohort of 80 out of 1,000 patients. Therefore, the sample size of this experiment is small. Pathophysiological characteristics are the foundation of the radiomics features. Since histopathological types and grades have more influence on image performance than genotypes in GC, this study limited the pathological type of GC to confirmed gastric adenocarcinoma at stage 3 or 4, excluding signet ring cell carcinoma or mucinous adenocarcinoma. The above criteria aimed to minimize the influence of factors other than DNA mismatch repair status on image performance.

In our study, radiomics signature comprised two robust radiomics features and manifest moderate predictive efficacy. Texture features consider the interaction between neighboring pixels and are therefore more propitious to quantifying tumor heterogeneity (41). The LASSO algorithm was used for feature redundancy elimination. This method has two primary preponderances. First, it allows features to be selected on the foundation of their univariable association with the outcome without overfitting. Next, it enables a signature to be constructed by a group of selected features (42). In this study, two texture features related to dMMR were selected to build the radiomics signature, which were intended to reveal tumor characteristics that are not apparent in the visual image (43). The two features were Original first-order Median and Original shape-Maximum 2D diameter Column. Original first-order Median is a first-order feature, while first-order statistics describes the distribution of voxel intensities within the image region defined by the mask through commonly used and basic metrics. The meaning of “median” in this context is the median gray-level intensity within the ROI. Original shape-Maximum 2D diameter Column is a shape feature. Maximum 2D diameter Column is defined as the largest pair-wise Euclidean distance between tumor surface mesh vertices in the row-slice plane. Using this approach, we attempted to develop a radiomics signature for the prediction of DNA mismatch repair deficiency in patients with GC. Our radiomics signature exhibited moderate discrimination, with an AUC of 0.81 in the training set and 0.78 in the internal testing set.

In this study, we extracted 2D CT annotations radiomics features based on single CT image slices. Meng et al. conducted a multicenter study comprehensively comparing the representation and discrimination capacity of 2D and 3D radiomics features regarding GC. The results based on three tasks showed that 2D and 3D models showed comparable ability to characterize GC. Their study indicated that 2D CT annotations might be a better choice than 3D in GC radiomics studies, because the latter may add noise (44).

Furthermore, the present study was not limited to the use of a single CT image slice. The importance of clinical characteristics should not be neglected, and the radiomics-derived data cannot predict all clinical decision problems. The univariate analysis showed that three clinical features (gender, age, and tumor location) were independent predictors. We then constructed the nomogram, a user-friendly, graphical analog computation device. The nomogram has clinical significance in the support of clinicians selecting individualized treatment for patients with GC. The AUC of the nomogram was 0.93, suggesting that the radiomics nomogram achieved greater predictive efficacy than either the radiomics signature or the clinical predictive model alone. The calibration and discrimination in the internal and external validation sets were also good. As a previous study revealed, dMMR GC typically has an antral location (45). Consistent with former research, the tumor locations in the present study were significantly different between the dMMR group and the pMMR group, with dMMR GC more likely to occur in the gastric antrum. The results of the present study also showed that pMMR (without DNA mismatch repair gene deficiency) was more likely to occur in men and at a younger age than the defective form. In contrast, Wang et al. reported that dMMR GC was more common in men (65% vs. 35%) (46) and that most of the cases were stage 2. In the present study, dMMR GC was more common in women, and this difference may be due to Wang’s study including mostly dMMR cases at stage 2, while the present study only included stage 3 or 4 GC patients.

The strength of our study is that the radiomics nomogram consists of only three clinical factors that are easily accessible preoperatively. Thus, the nomogram developed here may be used as a credible and non-invasive modality to preoperatively predict DNA mismatch repair deficiency in GC.

Our study was subject to some limitations. Firstly, the sample size of this study is small, including few patients with dMMR GC. Secondly, the tumor segmentation was manually sketched, which is time-consuming and laborious. In future work, computer algorithm-assisted automatic segmentation should be used. Thirdly, due to the retrospective nature of our study, selection bias was difficult to avoid, and patients not eligible for surgery were excluded. Fourthly, the slice thickness of most segmented CT images is 5 mm, and the volume effect of segmented CT images with a diameter of less than 5 mm is clear.

## Conclusions

In conclusion, our study demonstrated that the radiomics nomogram based on radiomics signature and clinical characteristics (age, sex, and tumor location) may be used for personalized preoperative prediction of DNA mismatch repair deficiency of GC and thereby assist in clinical decision-making.

## Data Availability Statement

The raw data supporting the conclusions of this article will be made available by the authors, without undue reservation.

## Ethics Statement

The studies involving human participants were reviewed and approved by the Cancer Hospital of the University of Chinese Academy of Science. Written informed consent for participation was not required for this study in accordance with the national legislation and the institutional requirements.

## Author Contributions

YT completed the initial manuscript and designed the whole study. JL and JC collected the clinical and imaging data and participated in revising the manuscript. CH collected the patients and recorded the needed information. ZX participated in its design. SD participated in the statistics and provided the result interpretation. XW helped collect the cases. RY helped collect the cases and reviewed the manuscript. XC revised the manuscript and guaranteed the entire study. All authors contributed to the article and approved the submitted version.

## Funding

This work was supported by the Key Laboratory of Prevention, Diagnosis and Therapy of Upper Gastrointestinal Cancer of Zhejiang Province (2022E10021) and the Medical Health Science and Technology Project of Zhejiang Province (2022KY655).

## Conflict of Interest

Author SD was employed by GE Healthcare.

The remaining authors declare that the research was conducted in the absence of any commercial or financial relationships that could be construed as a potential conflict of interest.

## Publisher’s Note

All claims expressed in this article are solely those of the authors and do not necessarily represent those of their affiliated organizations, or those of the publisher, the editors and the reviewers. Any product that may be evaluated in this article, or claim that may be made by its manufacturer, is not guaranteed or endorsed by the publisher.
